# The design and evaluation of the outflow structures of an interventional microaxial blood pump

**DOI:** 10.3389/fphys.2023.1169905

**Published:** 2023-05-11

**Authors:** Zhong Yun, Jinfu Yao, Liang Wang, Xiaoyan Tang, Yunhao Feng

**Affiliations:** School of Mechanical and Electrical Engineering, Central South University, Changsha, China

**Keywords:** microaxial blood pump, computational fluid dynamics, interventional surgery, flow field, hemolysis

## Abstract

Blood pump design efforts are focused on enhancing hydraulic effectiveness and minimizing shear stress. Unlike conventional blood pumps, interventional microaxial blood pumps have a unique outflow structure due to minimally invasive technology. The outflow structure, composed of the diffuser and cage bridges, is crucial in minimizing the pump size to provide adequate hemodynamic support. This study proposed four outflow structures of an interventional microaxial blood pump depending on whether the diffuser with or without blades and cage bridges were straight or curved. The outflow flow structure’s effect on the blood pump’s hydraulic performance and shear stress distribution was evaluated by computational fluid dynamics and hydraulic experiments. The results showed that all four outflow structures could achieve the pressure and flow requirements specified at the design point but with significant differences in shear stress distribution. Among them, the outflow structure with curved bridges would make the blood dispersed more evenly when flowing out of the pump, which could effectively reduce the shear stress at the cage bridges. The outflow structure with blades would aggravate the secondary flow at the leading edge of the impeller, increasing the risk of flow stagnation. The combination of curved bridges and the bladeless diffuser had a relatively better shear stress distribution, with the proportion of fluid exposed to low scalar shear stress (<50 Pa) and high scalar shear stress (>150 Pa) in the blood pump being 97.92% and 0.26%, respectively. It could be concluded that the outflow structure with curved bridges and bladeless diffuser exhibited relatively better shear stress distribution and a lower hemolysis index of 0.00648%, which could support continued research on optimizing the microaxial blood pumps.

## 1 Introduction

Heart failure is a multifaceted life-threatening syndrome with high morbidity and mortality that affects more than 64 million people worldwide (Savarese et al., 2022). Ventricular assist devices (VADs) have become a major treatment for patients suffering from heart failure in recent years (Han et al., 2018). However, the process of implanting VADs is time-consuming and laborious, leaving a large incision on the patient’s surface and posing an increased risk of infection (Ricklefs et al., 2018; Chatterjee et al., 2020). This problem is solved with the advent of percutaneous ventricular assist devices (pVADs), an advanced treatment with the advantages of rapid implantation, less invasive, low rates of complications, and easy nursing (Thiele et al., 2007; Chieffo et al., 2021). In addition to adapting to heart failure, pVADs can also provide hemodynamic support during the treatment of cardiogenic shock and high-risk PCI so as to achieve safe and complete revascularization (Kar, 2018).

The interventional microaxial blood pump is a kind of pVADs that can be rapidly implanted through minimally invasive surgery under the guidance and monitoring of imaging equipment, providing continuous blood flow from the left ventricle through the aortic valve to the ascending aorta (Pahuja et al., 2022). Due to the tiny size of the interventional blood pump, the impeller needs high enough speed to meet the clinical demand for pressure and flow (Christiansen et al., 2003; Koochaki and Niroomand-Oscuii, 2013). Excessive rotational speed of the impeller may lead to too much shear stress, increasing the risk of hemolysis and thrombosis (Yang et al., 2022). Moreover, there is also a problem of excessive shear stress at the outflow location because of the large flow rate of the blood after flowing out of the impeller (Yamane et al., 2021). Compared with the ordinary blood pump, the external diameter of the impeller of the interventional blood pump in this study was only 6 mm, which required a higher rotational speed when operating. This means that the problem of shear stress-induced hemolysis in the interventional blood pump is magnified that deserves to be studied in depth.

In recent years, the design and development of blood pumps have continued to work toward miniaturization and better biocompatibility (Schumer et al., 2016; Simon et al., 2019). To reduce the cycle and cost of the design and development process, computational fluid dynamics (CFD) has become an effective tool in blood pump research (Shukla et al., 2022). The CFD method enables accurate prediction of the internal flow field characteristics of the blood pump and assessment of the risk of hemolysis (Wu et al., 2017; Gross-Hardt et al., 2019). CFD studies of microaxial blood pumps have mainly focused on the effect of the impeller on pump performance characteristics and shear-induced hemolysis (Apel et al., 2001; Triep et al., 2006). However, the outflow structure is a design specific to maximizing the size of the blood pump, and its effect on blood damage and pump hydraulic performance remains unclear.

The purpose of this study was to analyze the specific effects of outflow structure on the performance of an interventional blood pump. Based on the proposed four outflow structure schemes, CFD was used to analyze the hydraulic performance, flow field, shear stress and hemolysis. Hydraulic testing was performed using a high-precision 3D-printed pump to verify the accuracy of the CFD method.

## 2 Matrials and methods

### 2.1 Pump design principle

In this paper, an interventional blood pump was developed to provide 0.5–4.5 L/min flow at a pressure ranging from 120 to 70 mmHg. Therefore, the design point chosen was to provide an average flow of 3.0 L/min at 100 mmHg. It should be noted that the classical axial flow pump design theory was proposed for large industrial pumps. Thus, there were definitely design theoretical limitations for a miniature blood pump with an impeller of only 6 mm. Even so, this classic design principle was the most relevant to the design of interventional blood pumps.

**FIGURE 1:**
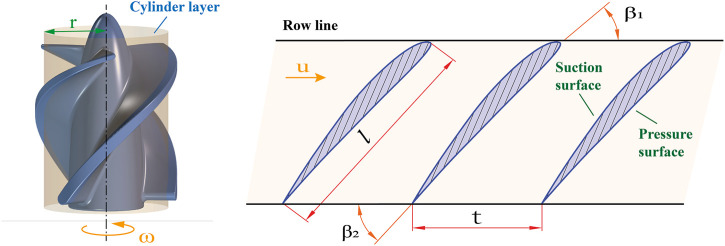
Expanded view of a cascade. $\beta_{1}/\beta_{2}$ are inlet and outlet relative angles; l is the chord length; t is the pitch; u is the tangential speed of impeller.

**FIGURE 2:**
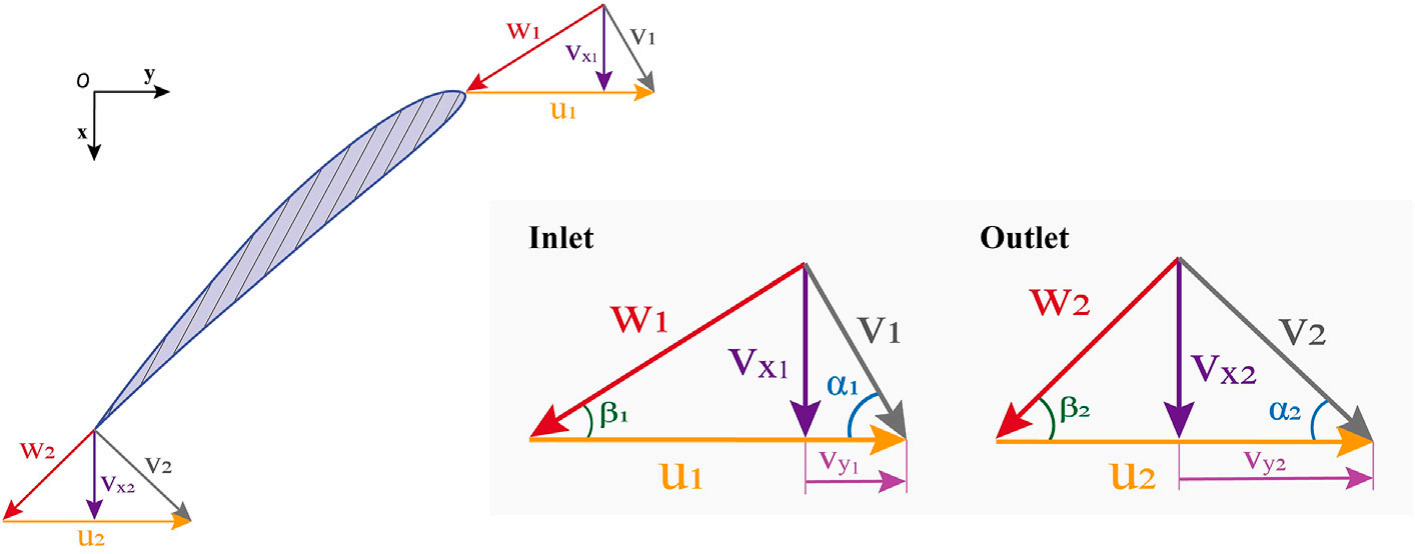
Impeller inlet and outlet velocity diagram. $W_{1}/W_{2}$ are relative velocities; $V_{1}/V_{2}$ are axial velocities; $U_{1}/U_{2}$ are tangential blade velocities.

The flow of blood in the pump was so complex that the method of cylindrical layer independence assumption was usually used in the design of axial flow pumps to simplify the calculation process. This assumption confined the flow in the flow passage to cylindrical sections of different diameters, ignoring the radial velocities of the liquid masses, and with no mutual interference between the cylindrical layers. The cylindrical layer intersected with the impeller surface to obtain a set of uniformly distributed blade profiles along the circumference called the cascade. The expanded view of a cascade is shown in Figure 1. The flow in the cascade passage of a microaxial blood pump was a plan flow, and the row line of the cascade was straight. The pitch of the cascade can be calculated by:

$$t=\frac{2\pi r}{Z}$$

where r is the radius of cylindrical layer, Z is the number of blades. An airfoil in the cascade was selected, and the velocity analysis method was used to study the motion law of the fluid in the flow passage. The impeller’s inlet and outlet velocity triangle was drawn, as shown in Figure 2. The flow of fluid particles in the impeller was a composite motion ( V=W+U ), and the plane composed of velocity triangles was tangent to the cylinder. Since the impeller diameter of the axial flow pump remained constant, the tangential blade velocity before and after the cascade was equal, i.e., $U_{1}/U_{2}$ . According to the continuity of the mass, and the constant cross-sectional area of the pump inlet and outlet, the axial velocity at the inlet ( $V_{x1}$ ) and outlet ( $V_{x2}$ ) could be obtained equally:

$$V_{x1}=V_{x2}=\frac{Q}{A}$$

where A is the cross-sectional area for flow, Q is the volume flow rate. The inlet and outlet relative angles of the impella can be calculated by:

$$\beta_{1}=\tan^{-1}\frac{V_{x1}}{U_{1}-V_{y1}}$$

$$\beta_{2}=\tan^{-1}\frac{V_{x2}}{U_{2}-V_{y2}}$$

where $V_{y1}$ and $V_{y2}$ are the tangential velocities of the fluid at the impella inlet and outlet. The angle of deflection caused by the blade acting on the fluid was defined as the difference between the inlet and outlet relative angle, i.e., $\Delta \beta =\beta_{2}-\beta_{1}$ . The larger the value of the deflection angle, the greater the work done by the impeller blades. The relative velocities at the inlet and outlet of the impeller can be obtained by:

$$W_{1}=\sqrt{{\left(U_{1}-V_{y1}\right)}^{2}+V_{x1}^{2}}$$

$$W_{2}=\sqrt{{\left(U_{2}-V_{y2}\right)}^{2}+V_{x2}^{2}}$$

The de Haller ratio was defined as the ratio of relative velocities at the inlet and outlet, i.e., $W_{2}/W_{1}$ . The smaller the de Haller ratio, the higher the blade loading. In order to avoid the flow separation on the suction side of the vane, resulting in pump performance degradation, we had to ensure that the de Haller should be greater than 0.7.

**FIGURE 3:**
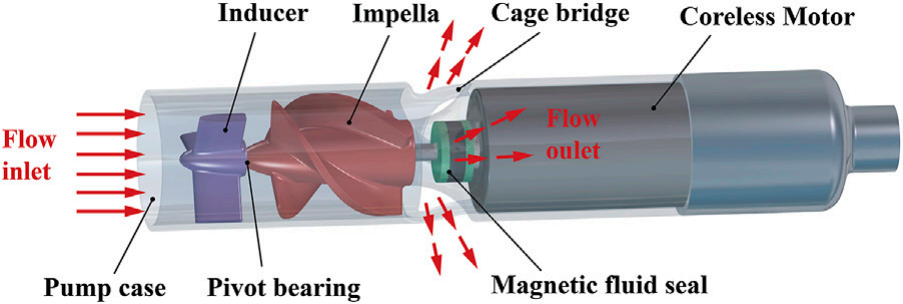
Conceptual design diagram of the interventional blood pump.

**FIGURE 4:**
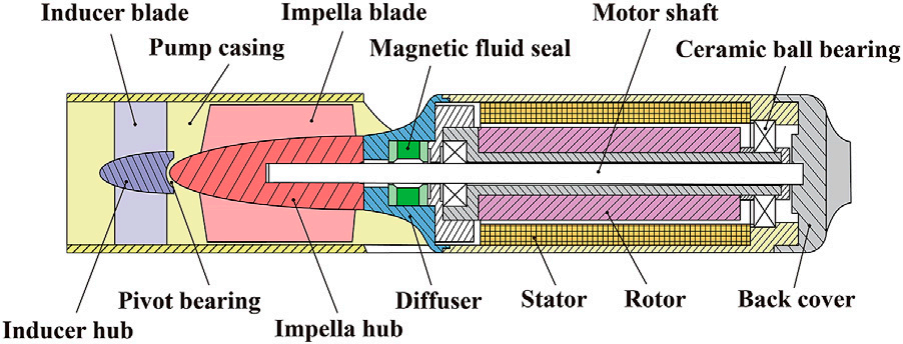
Schematic diagram of blood pump support structure scheme.

The conceptual design of the interventional blood pump is shown in Figure 3. The blood pump was mainly composed of three parts: the inducer, the impeller, and the outflow structure. Among them, the outflow structure consisted of a diffuser and three cage bridges. The primary function of the inducer was to make the blood flow more smoothly into the impeller. The outer diameter of the impeller was only 6 mm, and the radial gap between the impeller and the pump case was 0.1 mm. The impella was driven by the torque produced by a coreless motor, which might deliver a maximum speed of 33,000 r/min. Figure 4 illustrates the support structure scheme of the interventional blood pump. Two ceramic ball bearings were arranged on the motor shaft and played the leading supporting role. The magnetic seal acted not only as a seal to prevent blood from entering the motor, but also as an auxiliary support (Okamoto et al., 2022). A pivot bearing was designed at the front of the impeller to reduce the amplitude of the vanes and to avoid the problem of shear stress surge caused by the collision between the vanes and the pump casing.

**FIGURE 5:**
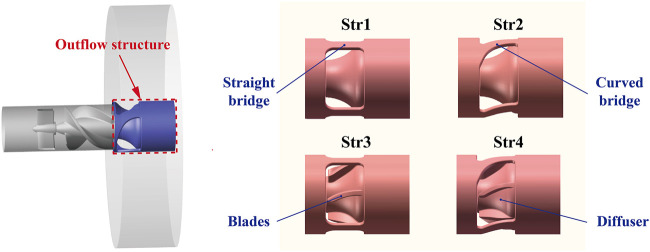
Four kinds of interventional blood pump outflow structures scheme diagram.

During the pump operation, the rotating impeller and stationary parts would produce relative motion, which might cause serious pressure pulsation (Al-Obaidi, 2020). The number of blades of the inducer and impeller and the number of bridges of the outflow structure was set to 3 to reduce the influence of rotor-stator interaction. As a component unique to the miniaturized design of blood pumps, the main function of the outflow structure was to disperse the blood while converting some of its rotational kinetic energy into pressure. In order to explore the influence of outflow structure on pump performance, this study proposed four preferable outflow structures of an interventional microaxial blood pump, as shown in Figure 5. Diffusers of Str1 and Str2 had no blades, while diffusers of Str3 and Str4 had blades. The cage bridges of Str1 and Str3 were straight, while the cage bridges of Str2 and Str4 were curved.

### 2.2 Computational fluid dynamics analysis

**FIGURE 6:**
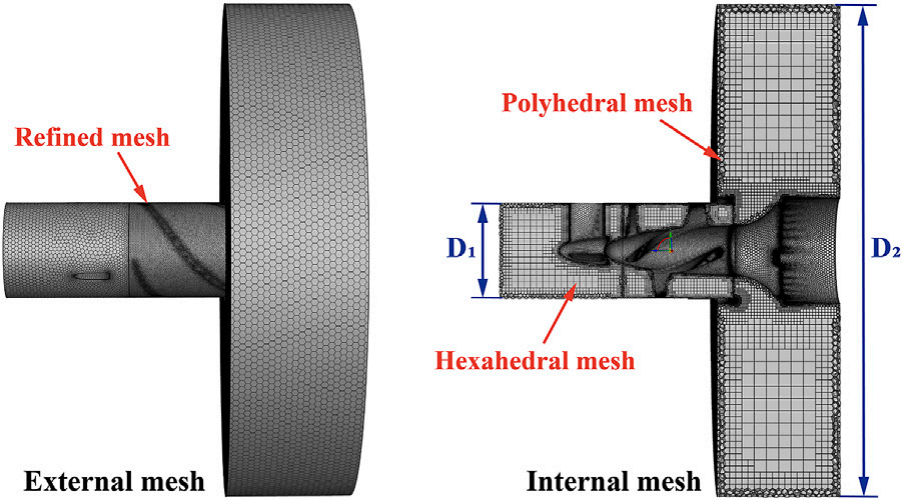
Schematic diagram of blood pump grid division.

After implantation into the heart, the interventional blood pump needed to be arranged through the heart valve, and the outflow structure was located in the ascending aorta. Therefore, in order to approximate the real implantation environment, the blood pump fluid domain was divided into the in-pump fluid domain and the outflow fluid domain. The geometric parameters of the outflow fluid domain were consistent with the ascending aorta internal diameter parameters to simulate the process of blood pumping from the outflow structure to the ascending aorta. For this study, Fluent Meshing (ANSYS Inc., United States) was used to establish the mesh of the blood pump fluid domain. In order to improve the solution efficiency and simulation accuracy, the latest Poly-Hexcore method was used for meshing. Figure 6 shows the schematic diagram of the blood pump grid division. In the figure, the $D_{1}$ value was 6.2 mm, indicating the diameter of the fluid domain’s inlet. The $D_{2}$ value was 32.0 mm (Huang et al., 2019), indicating the diameter of the fluid domain’s outlet. Specifically, polyhedral meshes were used to fill the near-wall surface, and hexahedral meshes were used to fill the core area. For impeller and outflow structures with a complex surface, refined meshes were set to realize local grid refinement. When the total number of grid nodes exceeded 1.48 million, and the grid in the impeller area accounted for 58%, the calculation results tended to be stable. Therefore, the total number of meshes for all blood pump models reached about 1.56 million.

Fluent 2021R2 (ANSYS Inc., United States) was used to perform CFD numerical simulations. The interventional blood pump was required to provide stable hemodynamic support to the patient. In order to obtain the state of physical quantities when the blood pump was operating steadily, the steady state condition was chosen for CFD simulations. As the working fluid of the blood pump, blood was simplified as an incompressible Newtonian fluid with a density of 1,050 $kg/m^{3}$ and a dynamic viscosity of 0.0035 $kg/\left(m∙s\right)$ in the simulation. The rotation of the impeller was realized by using a multiple reference system model. Besides, the RNG k - ε model was chosen for the turbulence model, which had good performance in calculating flow fields with large velocity gradients and strongly rotating flows. In order to improve the calculation accuracy near the wall, the enhanced wall treatment was adopted. The values of the boundary conditions were determined by the design point of the blood pump. The fluid domain’s inlet was designated as the Mass-Flow Inlet with a value of 0.0525 kg/s. The fluid domain’s outlet was defined as the Pressure Outlet with a value of 13,300 Pa. The SIMPLEC algorithm was selected to deal with the pressure velocity coupling to improve convergence speed. The number of iterations in the simulation was set to 500. When the residuals of the equations were all below $10^{-4}$ , the simulation was considered converged.

### 2.3 Hemolysis predictions

The shear stress generated by the high-speed rotation of the impeller was likely to damage the red blood cells in the blood and thus cause hemolysis, which was harmful to human physiological health. Scalar Shear Stress (SSS) in blood pumps was closely related to blood destruction, which could be expressed as follows:

$$\tau_{scalar}={\left[\frac{1}{6}\underset{i\neq j}{\sum }{\left(\tau_{ii}-\tau_{jj}\right)}^{2}+\underset{i\neq j}{\sum }{\tau_{ij}}^{2}\right]}^{1/2}$$

$$\tau_{ij}=\mu \left(\frac{\partial u_{i}}{\partial x_{j}}+\frac{\partial u_{j}}{\partial x_{i}}\right)$$

where μ is the fluid viscosity, i,j stand for X,Y,Z coordinate, u is the velocity. When red blood cells were subjected to shear stress of greater than 150 Pa, hemolysis occurs with the accumulation of exposure time (Thamsen et al., 2015). The power-law model proposed by Giersiepen et al. (1990) was used to assess the degree of damage to the blood.

$$HI\left(\%\right)=\frac{∆Hb}{Hb}\left(\%\right)=C∙\tau^{\alpha }∙t^{\beta }$$

where HI is the hemolysis index, ∆Hb represents the damaged hemoglobin concentration, Hb represents the total hemoglobin concentration, t is the exposure time. C,α,β are the constants obtained from the experimental data, $C=1.8\times 10^{-6}$ , α=1.991 , β=0.765 . Based on the above formula, the hemolytic index of the blood pump was predicted using the Lagrangian method. A total of 1,000 red blood cell particles with a diameter of 7μm were released from the inlet, and the particle trajectory was tracked. The predicted value of the cumulative hemolysis index to which each trajectory was subjected at a given moment can be calculated by the following equations:

$${HI}_{p,i}={HI}_{p,i-1}+\left(1-{HI}_{p,i-1}\right){hi}_{p,i}$$

$${hi}_{p,i}=1.8\times 10^{-6}∙{\tau_{\left(t_{i-1}\right)}}^{1.991}∙\left(t_{i}-t_{i-1}\right)$$

The average hemolytic index could be obtained by summing and averaging the ${HI}_{p}$ of N red blood cell particles:

$$HI=\frac{1}{N}\left(\sum_{p=1}^{N}{HI}_{p}\right)$$

### 2.4 Validations

**FIGURE 7:**
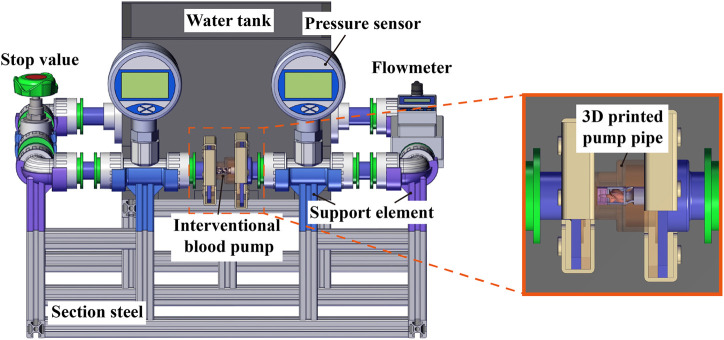
Schematic diagram of hydraulic cycle experiment platform.

**FIGURE 8:**
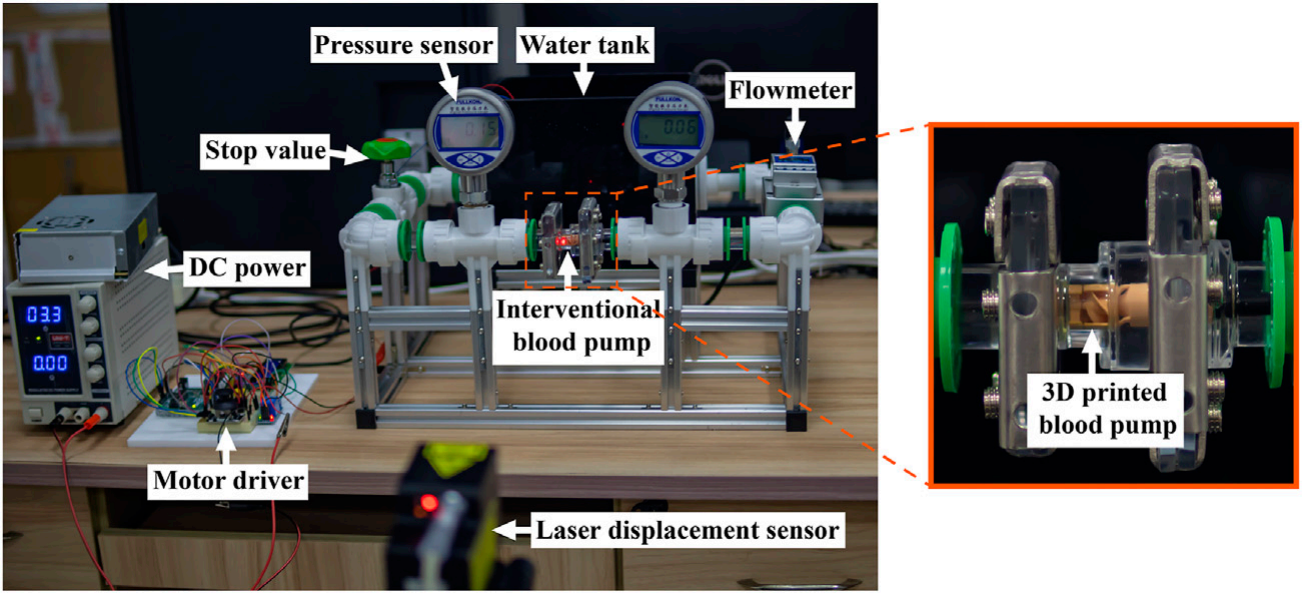
Hydraulic cycle experiment scene.

Hydraulic cycle experiments were performed and compared to the simulation data to verify the accuracy of the numerical simulation method. Figure 7 shows the schematic diagram of the experimental platform. The inlet and outlet pressure ports were measured by pressure sensors (FK-Y290, FULLKON, China). The flow rate was controlled by a stop value and measured by an ultrasonic flowmeter (DN20, XCON TECHNOLOGY, China). The photo of the experimental scene is shown in Figure 8. All structures of the interventional blood pump were made of German red wax 3D printing. The angular velocity of impeller rotation was measured by a laser displacement sensor (VSM1000, Julight, Italy). During the experiment, a glycerol-water solution with a mass ratio of 33% was used to simulate the working environment of the blood pump.

## 3 Results

### 3.1 Hydraulic performance

**FIGURE 9:**
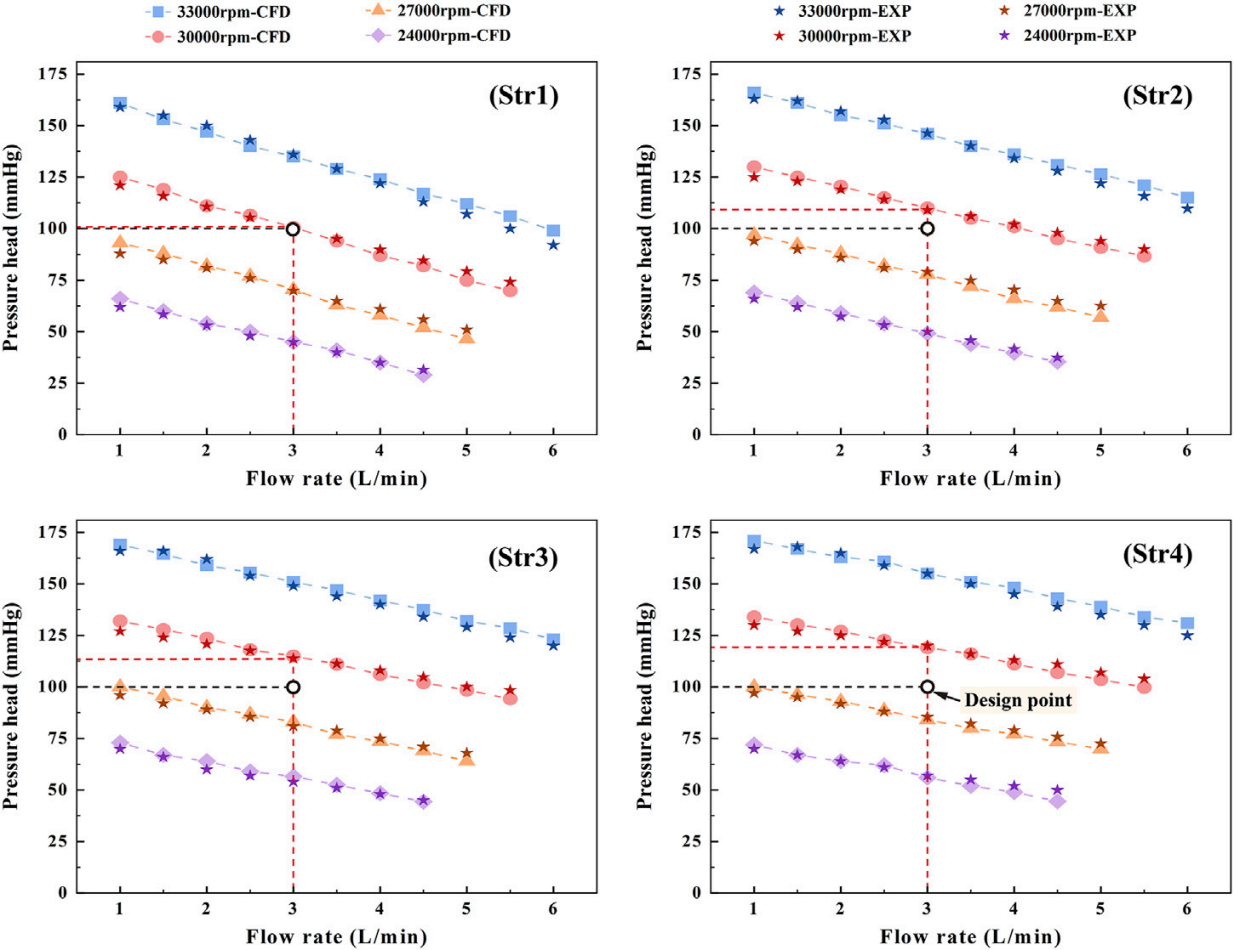
Head-flow curves obtained from CFD simulation and experimental validation.

Through CFD simulation and experimental validation, the head-flow curves were plotted for four-speed conditions, as shown in Figure 9. The design point of the interventional blood pump was specified at 3 L/min and 100 mmHg. Under the same speed conditions, the larger the head at a flow rate of 3 L/min, the better the hydraulic performance of the blood pump. At 3 L/min, the head of Str1 was 100.4 mmHg, Str2 was 109.8 mmHg, Str3 was 113.6 mmHg and Str4 was 120.2 mmHg. Compared with straight bridges, the design of curved bridges increased hydraulic efficiency by an average of 7.6%. Compared to the diffusers without blades, the design with blades increased the hydraulic efficiency by an average of 11.3%. When the flow and pressure required at the design point were reached, the rotational speeds of Str1∼Str4 were 29,960 r/min, 29,025 r/min, 28,804 r/min and 28,190 r/min, respectively. Reducing the impeller speed could help reduce the risk of hemolysis. Curved bridges and diffusers with blades could reduce the impeller speed at 775 r/min and 996 r/min, respectively. Comparing the results of CFD simulations and experimental measurements, the relative errors of the data were within 12%. The simulation method was proven reliable and could provide guidance for flow field analysis.

### 3.2 Flow field analysis

**FIGURE 10:**
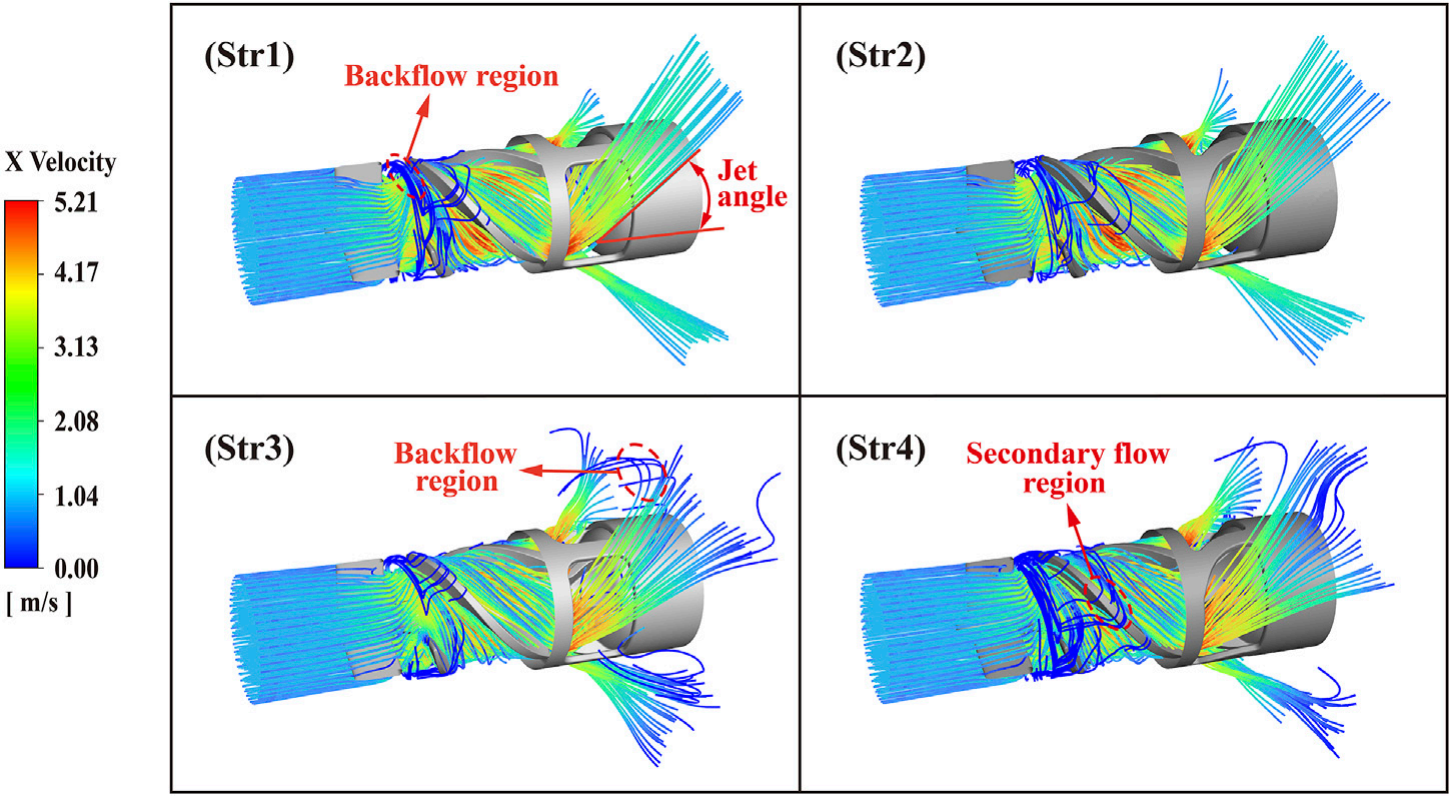
Velocity streamline diagram of blood pump outflow structures.

Figure 10 shows three hundred *x*-axis velocity streamlines passing through four outflow structures of the interventional blood pump. The blood flowed axially from the inlet and divided into three strands diagonally backwards from the outlet structure. The axial velocity reached a maximum when blood flowed out of the impeller, which indicated a greater risk of blood damage at the location of the outflow structure. Unsteady flow regions of backflow and secondary flow existed at the impeller’s leading edge and the tip clearance, respectively. Moreover, the diffuser with blades created a small backflow area after the blood flowed out of the pump. The percentage of unstable flow length in the Str1 streamline was 3.7%, Str2 was 2.1%, Str3 was 4.4%, and Str4 was 6.8%. Fewer backflow and secondary flow regions indicated a more stable internal flow, and Str2 had the relatively best velocity streamline. There were significant differences in the jet angles of the four outflow structures for blood flow, with Str1 at 38.5 ° , Str2 at 26.1 ° , Str3 at 18.6 ° , and Str4 at 9.3 ° . The smaller the jet angle, the better the hydraulic performance. Both curved bridges or diffusers with blades could reduce the angle of the jet.

**FIGURE 11:**
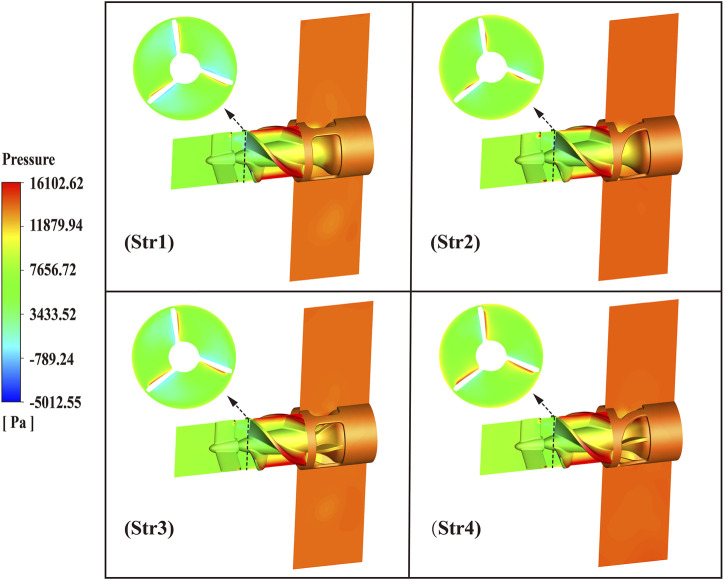
Pressure distribution diagram of blood pump outflow structures.

The pressure distribution of the four blood pump outflow structures is shown in Figure 11. From the figure, it could be seen that the pressure increased gradually along the direction of blood flow and was reasonably distributed. The blood pump could provide sufficient hemodynamic support only after the blood flowed out of the outflow structure. The average pressure at the cross-sectional of the leading edge of the impeller varied from 1923.0 Pa, 2406.8 Pa, 2593.7 Pa, and 2838.1 Pa for Str1 to Str4, respectively. The average cross-sectional pressure of the curved bridges was 16.1% higher than that of the straight bridges, and the diffusers with blades were 25.5% higher than that without blades. At the leading edge of the impeller, the flow was influenced by the pressure gradient, which could produce secondary flow and backflow. In order to evaluate the magnitude of the pressure gradient, the axial displacement of the leading edge cross-section was calculated for an average pressure increase of 1,000 Pa. The axial displacement of Str1 was 0.29 mm, Str2 was 0.34 mm, Str3 was 0.17 mm, and Str4 was 0.23 mm. The larger the axial displacement, the smaller the pressure gradient. Curved bridges reduced the pressure gradient by 23.9% compared to straight bridges. Diffusers with blades increased the pressure gradient by 36.5% compared to those without blades.

### 3.3 Shear stress analysis

**FIGURE 12:**
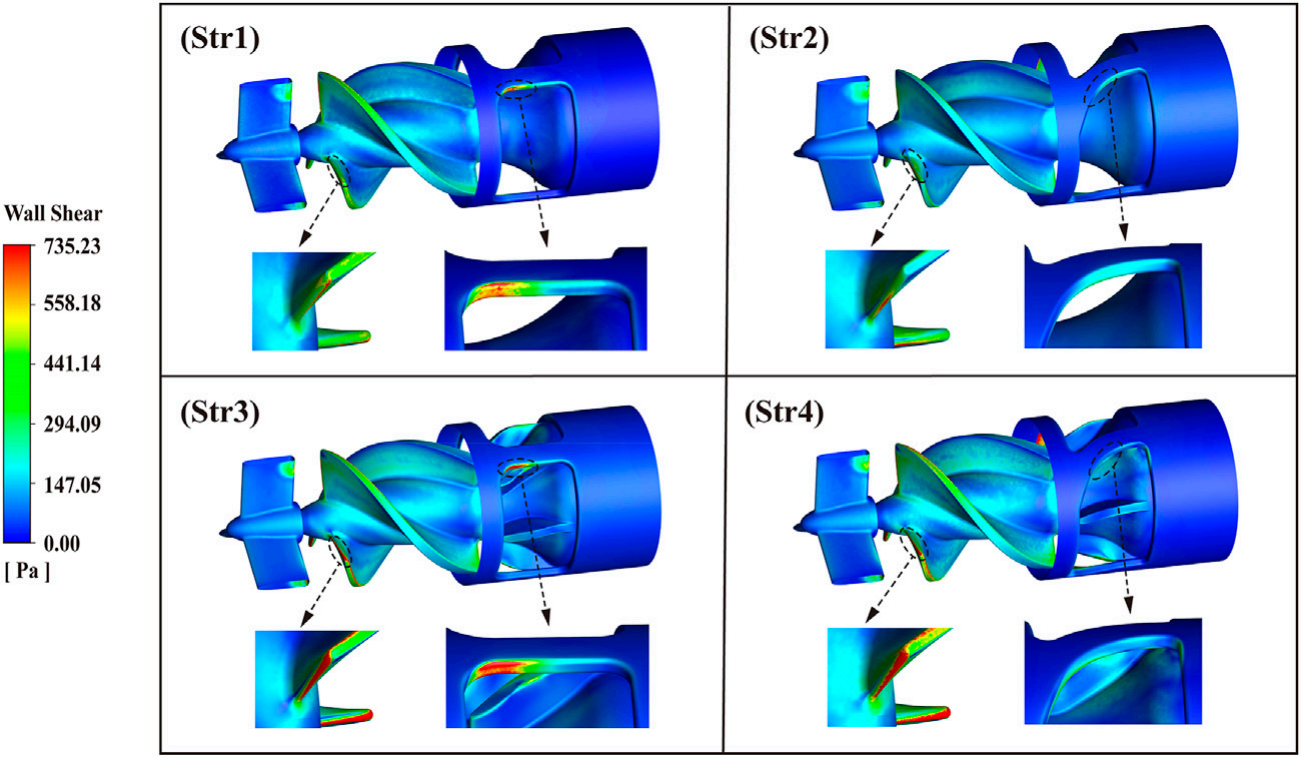
Wall shear stress distribution diagram of blood pump outflow structures.

**FIGURE 13:**
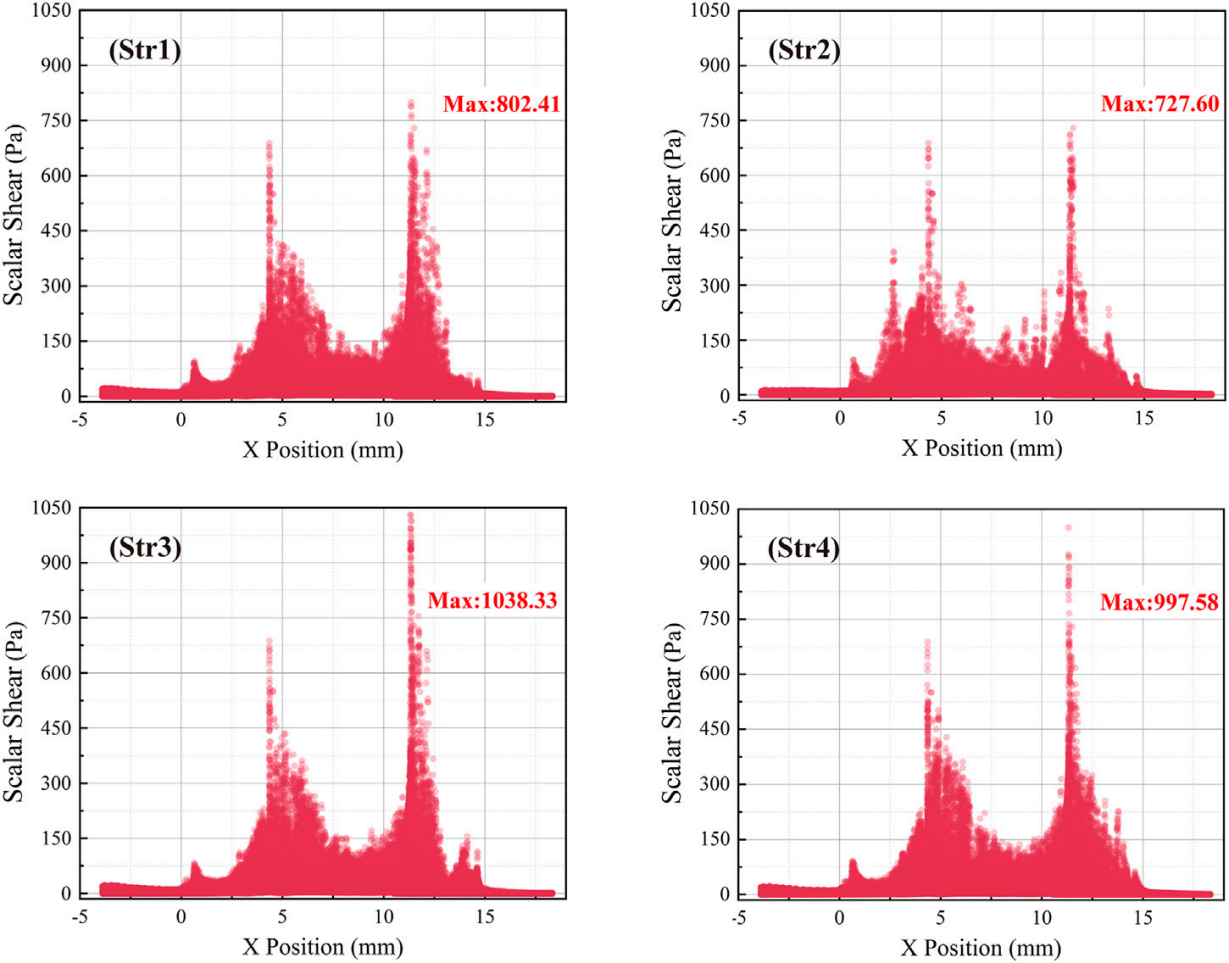
Scatter plot of scalar shear stress distribution along *x*-axis.

Wall shear stress (WSS) distribution of the interventional blood pump at the impeller speed of 30,000 r/min, as shown in Figure 12. Selected 735.23 Pa as the maximum value of the legend. WSS in most of the areas was below 200 Pa. The area of high WSS was mainly concentrated in three parts: The leading edge of the impeller, the top of the blade, and the bridges of the outflow structure. In particular, the blood flowed out of the impeller at a high velocity, and there was a more pronounced stress concentration problem at the straight bridge. After the diffuser was equipped with blades, not only the WSS in the impeller area was significantly increased, but also the average WSS of the outflow structure was increased. Data of 1,000 monitored RBC particles were calculated and extracted, and the scatter plot of the scalar shear stress distribution along the *x*-axis was drawn, as illustrated in Figure 13. The overall trend of SSS distribution was basically the same, with two peaks forming when the blood flowed into the impeller and out of the outflow structure. The first peak of the four outflow structures was almost the same, about 686 Pa. The second peak was the maximum value of SSS, with a significant difference. For Str3 and Str4 with increased diffuser blades, the values increased by 236 Pa and 270 Pa, respectively. Curved bridges could reduce the SSS at the outflow structure to a certain extent.

### 3.4 Hemolysis

Statistical analysis of the data is performed according to the platelet activation threshold of 50 Pa and the hemolysis threshold of 150 Pa, and the results are shown in Table 1. It could be seen that the lowest percentage of SSS greater than 150 Pa was Str2 with 0.26%, and the highest was Str3 with 0.37%. On the contrary, the lowest proportion of SSS below 50 Pa is Str3, with 96.52%, and the highest proportion was Str2, with 97.92%. Compared with the straight bridges, the outflow structure with curved bridges reduced SSS >150 Pa by 10.3% and increased SSS <50 Pa by 0.4%. Compared to the diffusers without blades, outflow structures with blades increased SSS >150 Pa by 26.3% and reduced SSS <50 Pa by 1.0%. After the removal of the blades by the diffuser, the distribution of SSS was more optimal, which meant that it had a lower risk of hemolysis and thrombosis. The size of the area of the high WSS region marked in red in Figure 12 had a similar law to the size of the percentage of high SSS. In the blood pump, the degree of blood damage was not only influenced by the magnitude of shear stress but also related to the exposure time of blood to shear stress. Matlab was used to calculate the simulation results and obtain each outflow structure’s average hemolysis index (HI). The calculated HI for Str1 was 0.00725%, for Str2 was 0.00648%, for Str3 was 0.00839%, and for Str4 was 0.00805%. The higher the hemolysis index, the higher the degree of hemolysis, with the highest HI value of Str3 being 29.5% higher than the lowest Str2. Compared with straight bridges, the design of curved bridges reduced the HI value by an average of 7.1%. Compared to the diffusers without blades, the design with blades increased the HI value by an average of 19.7%. In terms of predicted results, Str2 showed a relatively better anti-hemolysis performance. The scheme of curved bridges without blades could reduce the hemolysis degree of the interventional blood pump.

**TABLE 1:** Comparison of SSS volume fraction and hemolysis index of blood pump outflow structures.

Blood pump	SSS>150 Pa (%)	SSS<50 Pa (%)	Hemolysis index (%)
Outflow Str1	0.31	97.46	0.00725
Outflow Str2	0.26	97.92	0.00648
Outflow Str3	0.37	96.52	0.00839
Outflow Str4	0.35	96.83	0.00805

## 4 Discussion

In this research, four outflow structures composed of cage bridges and diffusers were designed and analyzed for the structural characteristics of the interventional blood pump. The *in vivo* conditions of the blood pump mainly included size, pressure and flow requirements. Clinically, for microaxial blood pumps with an outer diameter of approximately 7 mm, the main implantation was via the axillary artery using a 10 mm Dacron vascular graft (Anderson et al., 2021; Shugh et al., 2021). The outside diameter of the interventional blood pump designed in the paper was 6.5 mm, which could meet the clinical size requirements. When the blood pump was implanted in the patient, it was required to provide 100 mmHg mean aortic pressure at a coronary flow rate of 3 L/min. The results of head-flow curves showed that the pressure heads of Str1 to Str4 were 100.4 mmHg, 109.8 mmHg, 113.6 mmHg and 120.2 mmHg, respectively, when the blood pump provided a steady flow of 3 L/min at 30,000 r/min. The suggested safe operating region for blood pumps was to provide at least 87.5 mmHg pressure head demand at 3 L/min (Smith et al., 2018). If the pressure head provided by the interventional blood pump was lower than 87.5 mmHg, the retrograde flow might occur, causing damage to the heart valve. Thus, all four outflow structures were able to provide stable mechanical circulatory assisted support to the patient without the risk of blood retrograde flow.

From the velocity flow diagram, it can be seen that the diffuser equipped with blades generated a small amount of backflow at the outlet position and exacerbated the secondary flow and backflow at the leading edge of the impeller. This phenomenon was caused by changes in the pressure gradient inside the blood pump and was normal (Zhu et al., 2010). Combined with the pressure distribution diagram, we can observe that the pressure change in the area where the backflow occurs was noticeable. For blood pumps, on the one hand, backflow increased pump disturbance and amplifies vibration problems. On the other hand, backflow increased the time erythrocytes are exposed to shear stress, boosting the risk of hemolysis (Telyshev et al., 2018). Specifically, the design of the curved bridges enabled a more even pressure distribution inside the pump, with fewer backflow problems.

During the development of blood pumps, exploring the risk of shear stress-induced hemolysis is the primary means of judging good or lousy biocompatibility (Chan et al., 2017). The high shear region of the interventional blood pump was concentrated on the impeller’s leading edge and the outflow structure’s bridges. Although the pressure change was equally significant at both locations, the considerable difference in flow rate could be the leading cause of the high shear stress. When blood entered the impeller, the flow velocity magnitude and direction changed significantly. Similarly, when blood flowed through the outflow structure, the fluid flowed diagonally backward from three strands, and the flow velocity also changed significantly. On the scatter diagram of shear stress, the two regions mentioned above formed two peaks due to their high values. Moreover, the blood flowed faster through the outflow structure, so the shear stress was at its maximum at this position. This maximum value decreased for the model with curved bridges because the curved bridges allowed a more uniform dispersion of blood flowing through the outflow structure. Statistical data showed that the magnitude of the hemolysis index was positively correlated with the percentage of high shear stress. Therefore, there might be more significant shear stress and a higher risk of hemolysis at positions where the flow rate was more variable. It was worth noting that the outflow structure with blades had a higher hemolysis index. This was because the diffuser with blades could reduce the flow loss and produce higher pressure output. In other words, it resulted from coupling both flow rate and pressure gradient.

Simulation results were compared using the microaxial blood pump HMII and Sputnik1 to verify the plausibility of the hemolysis prediction. (Thamsen et al., 2015; Romanova et al., 2022). The HMII had a rotor diameter of 12 mm, and its hemolysis index HI at 15,000 r/min was 0.00375%. Sputnik1 had a rotor diameter of 16 mm, and HI value at 9,100 r/min was 0.0090%. The interventional blood pump studied in this paper had a rotor diameter of only 6 mm and an operating speed of 30,000 r/min. According to the results of hemolysis prediction, Str2 had the lowest HI of 0.00648%, and Str3 had the highest HI of 0.00839%. Higher HI values indicate poorer hemolysis performance of the blood pump. Compared with the blood pump in the above study, the hemolysis performance of the interventional blood pump was within acceptable limits.

There are some limitations to this research. In hydraulic experiments, we verified the results of the numerical simulations using a high-precision 3D-printed blood pump model. In fact, the material of the clinically used blood pump is titanium alloy, which is prepared by five-axis machining. This study was only a preliminary validation of the biocompatibility of the interventional blood pump, focusing on shear-induced hemolysis. However, the assessment of blood damage is complex, and *in vitro* hemolysis tests will be required to validate the results’ accuracy further. Moreover, the numerical simulations need to be further investigated to verify the plausibility of the Poly-Hexcore meshing method and the RNG k - ε turbulence model.

## 5 Conclusion

In this study, four outflow structures of the interventional blood pump were proposed and numerically simulated using CFD methods. The simulation calculation results show that all four outflow structure solutions can achieve the designed hydraulic performance requirements. The design of curved bridges and diffusers with blades can improve hydraulic performance by 7.6% and 11.3%, respectively. However, there are significant differences in the effects of the two on shear stress distribution. Outflow structures with curved bridges will reduce high shear stresses (>150 Pa) by 10.3% and reduce the hemolysis index by 7.1%. Conversely, outflow structures with blades will increase high shear stress (>150 Pa) by 26.3% and increase the hemolysis index by 19.7%. Besides, the combined scheme of curved bridges without blades has relatively better shear stress distribution and a lower hemolysis index of 0.00648%, which is a relatively better scheme.

## Data Availability

The original contributions presented in the study are included in the article/supplementary material; further inquiries can be directed to the corresponding authors.
